# Autoimmune-mediated astrocytopathy

**DOI:** 10.1186/s41232-023-00291-5

**Published:** 2023-07-18

**Authors:** Makoto Kinoshita, Tatsusada Okuno

**Affiliations:** Department of Neurology, Osaka University Graduate School of Medicine, 2-2 Yamadaoka, Suita, Osaka 565-0871 Japan

**Keywords:** Astrocyte, AQP4, Autoimmunity

## Abstract

Recently accumulating evidence identified the disease entity where astrocytes residing within the central nervous system (CNS) are the target of autoantibody-mediated autoimmunity. Aquaporin4 (AQP4) is the most common antigen to serve as astrocyte-targeted autoimmune responses. Here, in this review, the clinical and pathological aspects of AQP4-mediated astrocyte disease are discussed together with the pathogenic role of anti-AQP4 antibody. More recently, the mechanism of immune dysregulation resulting in the production of astrocyte-targeted autoantibody is also revealed, and the postulated hypothesis is discussed.

## Background

Among the autoimmune-mediated disease of the central nervous system (CNS), multiple sclerosis (MS) has been recognized as the most prevalent inflammatory disease in the field of neurology [1]. In contrast to the distinct demyelinating lesions characteristic to MS [1], during the past decade, there has been huge progress regarding the autoimmunity targeting astrocytes, coined as neuromyelitis optica spectrum disorder (NMOSD) [2]. NMOSD is characterized by the appearance of specific autoantibody against the water channel, aquaporin-4 (AQP4) [2], expressed on the endfeet of astrocytes. Ever since the discovery of the anti-AQP4 antibodies (Abs), there has been huge progress in our understanding of its pathogenicity to induce astrocyte dysfunction and subsequent inflammation that takes place within the CNS [2]. Here in this review, the clinical and pathological characteristics of NMOSD and the pathogenic mechanism of astrocyte-targeted autoimmunity are discussed.

### Astrocyte-targeted autoimmunity

Ever since the discovery of the disease-specific antibody against the water channel expressed on the endfeet of astrocytes [3, 4], anti-AQP4 antibody, the disease entity of NMOSD has now widely been acknowledged as one of the major inflammatory diseases of the CNS [2]. The clinical symptoms of NMOSD were initially recognized to be optic neuritis and myelitis [5]; however, with the accumulation of clinical evidence of the variety of the lesions manifested in patients, currently typical syndromes of NMOSD encompass area postrema syndrome [6], brain stem symptoms, and hypothalamic dysfunctions [7]. The prevalence of NMOSD is higher in non-White populations, and epidemiological studies show the average onset of the disease is approximately 41.1 years with female predominance [5, 8]. The radiological features of NMOSD are marked by longitudinally extensive transverse myelitis (LETM), where lesions extend more than 3 vertebral segments [7]. In contrast to the attack of MS, the acute symptoms of NMOSD mostly show more severe symptoms with relatively less marked response to the initial treatment with corticosteroids, highlighting the highly destructive nature of the disease activity [7].

Anti-AQP4 Ab is a highly specific and sensitive disease marker of NMOSD [3, 4] and is mostly detected with the serum of the patient and occasionally also within the cerebrospinal fluid (CSF) [9]. Several studies performed in vitro and in vivo as described in later sections showed that anti-AQP4 Ab is pathogenic autoantibody and serves as the core in the pathogenic mechanism of NMOSD [10–12]. In this regard, patients with NMOSD are shown to benefit from plasmapheresis during the acute attack of the disease [13], and furthermore, B cell depletion therapy is shown to be efficacious for the prevention of further relapses [14].

### Pathological aspects of NMOSD

In contrast to the distinct demyelinating lesions observed in MS [1], the pathological features of NMOSD are marked by the destruction of astrocytes. The relative loss of glial fibrillary acidic protein (GFAP) compared to preservation of myelin basic protein was reported in the study of immunostaining of spinal cord lesions of NMOSD patients [15]. The lesions of NMOSD are also marked by perivascular deposition of immunoglobulins and activated complement complex [15]. The acute lesions of NMOSD are also highly destructive in line with the severe clinical course after the development of attacks. More recently, the astrocyte damage has been more extensively analyzed according to the chronological time frame [16]. These observations highlight that astrocytopathy is the core mechanism during the development of the disease.

### Anti-AQP4 antibody

AQP4 is highly expressed at the endfeet of astrocytes and modestly on ependymal cells with the CNS [17]. AQP4 is membrane-spanning protein expressed on the surface of astrocytes, thus easily accessible to anti-AQP4 Ab which enters into the CNS. AQP4 forms a tetramer as a minimum unit [18], and furthermore, it can also change in the form of supramolecular aggregates, called orthogonal arrays (OAPs) [19, 20]. Two isoforms of AQP4, M1 and M23, exist, and M23 is known to be capable of establishing larger OAPs [21–23]. It has been shown that anti-AQP4 Abs present within the patients with NMOSD preferentially binds to AQP4 in the form of OAPs. There are several reports that show the anti-AQP4 Ab binding to AQP4 expressed in cell lines results in endocytosis of AQP4 [24].

### The pathogenicity of anti-AQP4 antibody

Several studies have demonstrated the pathogenicity of anti-AQP4 Ab both in vitro and in vivo [10–12, 25]. The most compelling evidence of anti-AQP4 Ab was shown in animal models of passive transfer [10–12]. To overcome the issue that anti-AQP4 Ab is mostly not accessible to CNS without the breakage of blood-brain barrier (BBB), several groups have utilized EAE as the recipient of systemically administered patient-derived antibody [10–12]. The remarkable findings of these models were that the animals that received anti-AQP4 Ab containing immunoglobulins recapitulated the pathological features of NMOSD pathology of the patients’ lesions [10–12]. The lesions formed in the spinal cord of passive transfer models showed loss of GFAP and AQP4 in immunostaining, accompanied by perivascular deposition of immunoglobulins and activated complement, C5b-9 [10]. Further studies involved models where direct injection of anti-AQP4 Ab to the brain was utilized. When anti-AQP4 Ab was injected intracerebrally into rat, marked loss of GFAP and AQP4 was observed with infiltrating macrophages and granulocytes [26].

These observations were compelling to conclude the pathogenicity of anti-AQP4 Ab and established the pivotal role of anti-AQP4 Ab in the pathogenic mechanism of NMOSD [27]. Although these models utilized the strategy so that anti-AQP4 Ab can get access to CNS irrespective of the presence of BBB, in patients with NMOSD, CNS-infiltrating T cells are also considered to be playing important role to breach the integrity of BBB [9], thus making anti-AQP4 Ab cross the border into the CNS.

### Mode of anti-AQP4 Ab action

**Fig. 1:**
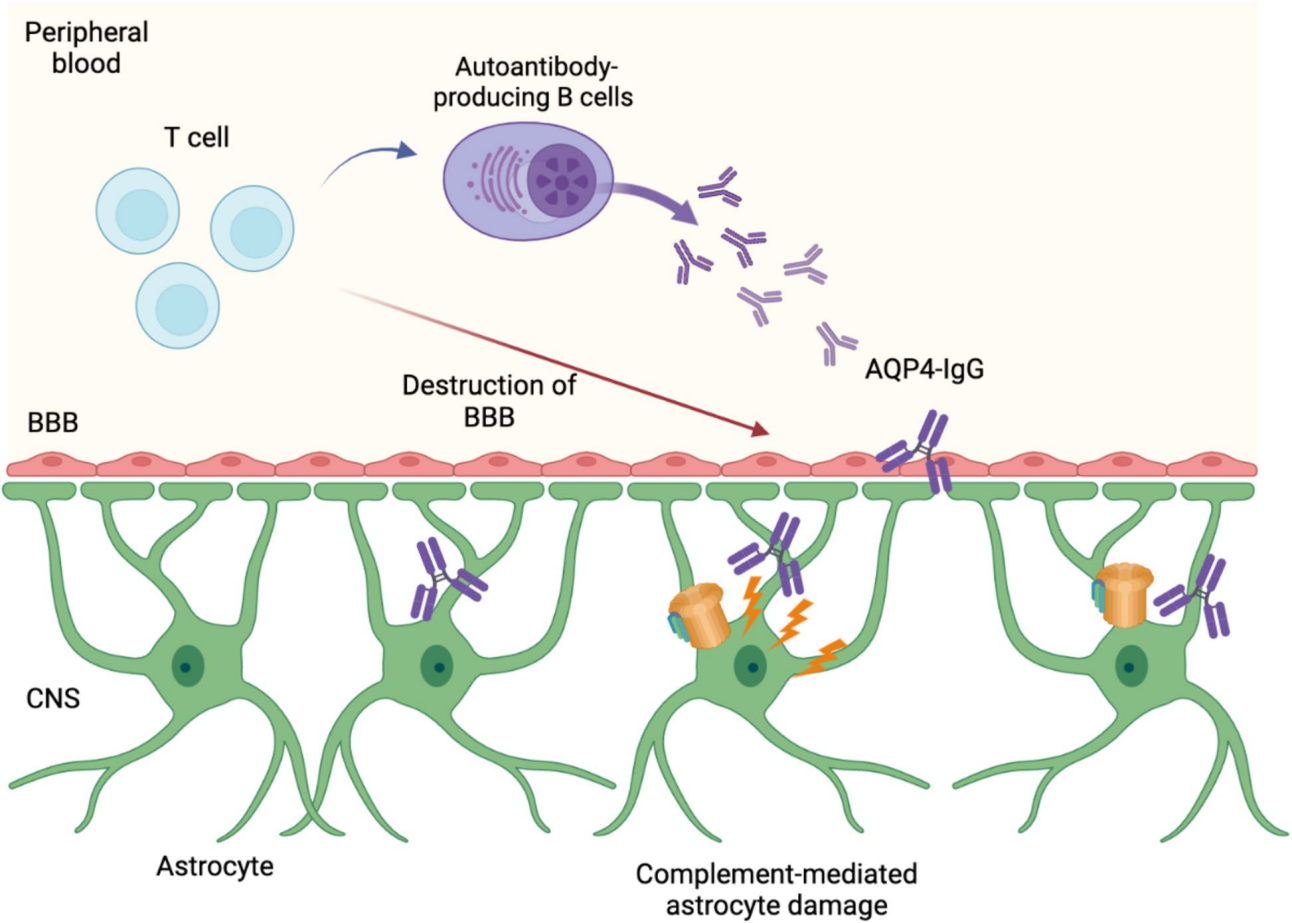
The pathogenicity of AQP4-IgG. The destruction of BBB induced by CNS-infiltrating T cells allows AQP4-IgG produced by B cells in peripheral blood to get an access to AQP4 expressed on astrocytes, leading to complement-dependent astrocyte damage. Figures were created with biorender.com

With regard to the mode of actions that anti-AQP4 Ab exerts its pathogenic effect, both complement-dependent cytotoxicity (CDC) and antibody-induced cellular cytotoxicity (ADCC) are proposed [27]. As mentioned above, pathological hallmark of NMOSD lesions is the deposition of complement, and the cellular necrosis of astrocytes by anti-AQP4 Ab has also been shown by in vitro studies [25]. Large proportion of anti-AQP4 Ab present in the serum of the patients belongs to IgG1 subclass, thus indicating that CDC is induced following the classical complement pathway (Fig. 1). Further evidence of complement involvement in the pathogenesis of NMOSD is also proven by high efficacious treatment of anti-C5 monoclonal antibody for the prevention of relapses of NMOSD patients in clinical trials [28]. Although it remains to be clarified to what extent ADCC is actually playing role in establishing the lesion formation in patients, studies indicate that Fc region of anti-AQP4 Ab binds to Fc gamma receptors, thus leading to activation of NK cells [29, 30, 27]. In addition, recent report utilizing human astrocytes developed using induced pluripotent stem cells showed structural alterations of mitochondria caused by antibodies derived from NMOSD patients [31].

### Microglial involvement in NMOSD

Despite the fact astrocytes are the primary types of cells that are injured in the pathogenesis of NMOSD, recent report also shed light on the role of microglia in accelerating the lesion formation [32]. It is postulated that anti-AQP4 Ab induce C3 production by astrocytes, leading to activation of microglia through C3a receptor. Activated microglia can further secrete C1q to exacerbate the complement-dependent cytotoxicity in NMOSD lesion or either secrete various types of proinflammatory cytokines [33]. Interestingly, patients with LETM of NMOSD frequently suffer from intractable neuropathic pain in the chronic phase of the disease [34]. Microglia is considered the major type of cells for establishing neuropathic pain in general [35], and IL-6 has been shown to have the capacity to activate microglia in vitro [36]. The clinical relevance of microglial involvement in the pathogenesis of NMOSD is further supported by the fact that anti-IL-6R monoclonal antibody relieve to some extent the neuropathic pain of NMOSD as reported in several studies [37]. In addition to IL-6, recently, it was shown that ATP is released from dying astrocytes exposed to anti-AQP4 Ab, inducing neuropathic pain in vivo [34]. Several reports have demonstrated the pivotal role of purinergic signaling and microglial activation involved in the development of neuropathic pain [38]. Thus, the distinct roles of IL-6 and ATP in neuropathic pain observed in NMOSD patients remain to be elucidated in future studies.

### NMOSD and peripheral immune signature of type 1 interferon

**Fig. 2:**
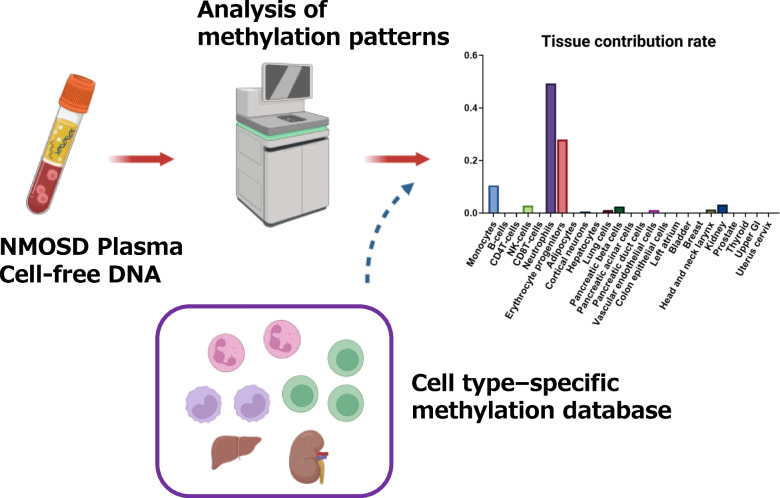
Identification of the source of cell-free DNA by cell type-specific methylation patterns. Figures were created with biorender.com

With accumulating evidence that anti-AQP4 Ab plays central role for the induction of astrocyte autoimmunity in NMOSD, it is essential to clarify the immune mechanism that leads to the production of the autoantibody in peripheral immune system. In this regard, it is of note that patients with NMOSD occasionally coexist with other autoimmune diseases, such as systemic lupus erythematosus (SLE) or Sjogren’s syndrome (SS), where type 1 interferon (IFN) pathway is suggested to be underlying as the background immune signature [39]. More importantly, patients with NMOSD are well known to show exacerbation of the disease activity in cases IFN-β, the disease-modifying drug approved for MS, is applied. Recent study showed that type 1 IFN stimulates the production of IL-6 from B cells, thus accelerating the Th17-mediated autoimmunity [40]. In addition, peripheral blood mononuclear cells (PBMCs) derived from patients with NMOSD show higher expression of type 1 IFN compared to healthy donors [41]. It has been shown that cell-free DNA (cfDNA) within the sera of NMOSD is capable of inducing mRNA expression of type 1 IFN [41]. Most importantly, the analysis of cfDNA methylation pattern of NMOSD has led to the identification of neutrophils in the peripheral circulation as the major source of cfDNA release in NMOSD patients [41] (Fig. 2). This discovery is further supported by the results of whole blood transcriptome analysis of NMOSD patients, where neutrophil activation pathway was shown to be the primary immune signature representing the immune dysregulation of NMOSD [41].

## Conclusions

With the discovery of astrocyte-specific pathogenic autoantibody in patients with NMOSD, the detailed mechanism leading to astrocyte destruction has been recently revealed. The questions remain whether astrocyte functions are preserved during the remission phase of the disease. Furthermore, the chronic reconstitution of CNS resident cells after astrocyte injury of acute attack is another issue to be addressed in future study. In this regard, in vivo imaging technology to elucidate the function of CNS resident cells in patients with NMOSD would serve as a pivotal approach to provide deeper insight into the broader picture of NMOSD pathogenesis.

## Data Availability

Not applicable

## Abbreviations

CNS: Central nervous system
AQP4: Aquaporin4
MS: Multiple sclerosis
NMOSD: Neuromyelitis optica spectrum disorder
Abs: Antibodies
LETM: Longitudinally extensive transverse myelitis
CSF: Cerebrospinal fluid
GFAP: Glial fibrillary acidic protein
OAPs: Orthogonal arrays
BBB: Blood-brain barrier
CDC: Complement-dependent cytotoxicity
ADCC: Antibody-induced cellular cytotoxicity
SLE: Systemic lupus erythematosus
SS: Sjogren’s syndrome
IFN: Interferon
PBMCs: Peripheral blood mononuclear cells
cfDNA: Cell-free DNA
